# Incidence and risk factors for unplanned readmission after total hip arthroplasty for osteonecrosis of the femoral head

**DOI:** 10.3389/fsurg.2024.1408343

**Published:** 2024-11-29

**Authors:** Meng Wang, Xuemei Yang, Junyong Li, Chengsi Li, Yulong Zhang, Xuewei Hao

**Affiliations:** ^1^ Department of Orthopaedic Surgery, Shijiazhuang People’s Hospital, Shijiazhuang, Hebei, China; ^2^ Obstetrics Department, The Fourth Hospital of Shijiazhuang, Shijiazhuang, Hebei, China; ^3^ Department of Orthopaedic Surgery, Hebei Medical University Third Hospital, Shijiazhuang, Hebei, China

**Keywords:** osteonecrosis of the femoral head, hip replacement, unplanned readmission, clinical characteristics investigation, risk factors

## Abstract

**Objective:**

To investigate the incidence, primary causes, and risk factors for unplanned readmissions within one year after the first primary total hip arthroplasty (THA) for osteonecrosis of the femoral head (ONFH).

**Methods:**

Data were retrospectively collected from patients who had undergone the first primary THA for ONFH at two tertiary hospitals between January 2021 and December 2022, with complete 1-year follow-up assessments. Patients who experienced an unplanned readmission within 1 year were classified as the readmission group, while the others as the non-readmission group. The incidence rate and primary causes of unplanned readmission were determined, and the risk factors were identified through univariate and multivariate analyses.

**Results:**

A total of 594 eligible patients were included, with 363 being men (61.1%) and an average age of 59.2 years at the time of surgery. Forty-seven patients were readmitted within one year, representing an accumulated rate of 7.9%. Among these, 18 (38.3%) readmissions occurred within 30 days and 27 (57.4%) within 90 days. The primary reasons for readmissions included hip dislocation (35.3%), followed by periprosthetic fracture, deep vein thrombosis, delayed incision healing, surgical site infection and others. The multivariate regression model revealed that age (every 10-year increment, OR, 1.39; 95% CI, 1.12–1.88), ARCO stage IV vs. Ⅲ (OR, 3.72; 95% CI, 1.96–7.22), CCI ≥4 vs. <4 (OR = 5.49; 95% CI, 2.16–13.77), admission anemia (OR, 2.72; 95% CI, 1.37–6.83) and surgeon inexperience (OR, 2.74; 95% CI, 1.29–6.73) were significantly associated with unplanned readmission.

**Conclusions:**

These findings provide valuable clinical insights into unplanned readmission after THA for ONFH and may aid in preoperative counselling for patients and enhance perioperative care.

## Introduction

Osteonecrosis of the femoral head (ONFH) is a debilitating condition characterized by severe hip joint dysfunction, with its global incidence on the rise, currently affecting more than 20 million individuals worldwide (1, 2). A significant proportion of those affected are relatively young, typically between the ages of 30 and 50 years, and often serve as the primary breadwinners for their families (1). For patients with end-stage ONFH (Association Research Circulation Osseous, ARCO III and Ⅳ), total hip arthroplasty (THA) is recognized as the only effective treatment option (3). However, the high rate of postoperative complications remains a major concern. Especially, patients who experience major postoperative complications requiring readmission for medical or surgical interventions will face considerable morbidity and substantial healthcare costs (4).

In recent years, clinical studies have increasingly focused on readmission rates following THA for ONFH. Sodhi et al. (5) reported a 2.3-fold significantly increased risk of 30-day readmission for ONFH patients compared to those without, based on a retrospective analysis of 8,344 matched patients undergoing THA. Similarly, Sax et al. (4) found that patients undergoing THA for ONFH had a significantly higher readmission rate at 90 days (13.1% vs. 7.7%) and an 11.7% increase in hospitalization costs compared to those treated for osteoarthritis, utilizing a large dataset from a national readmissions database. However, the readmission rates varied considerably, with a 30-day rate ranging from 1.6% to 6.1%, and a 90-day rate from 2.6% to 13.1% (4–8). Potential reasons for this variability may be the relatively small sample size, heterogenous populations, different follow-up periods, and the various methodologies employed. Furthermore, very few studies have examined the potential risk factors associated with readmission and identified the primary causes (6, 9), making it challenging to understand the detailed clinical characteristic of this issue. More recently, Wang et al. (6) conducted a study involving 876 patients and reported a 1-year readmission rate of 4.7%. They identified advanced age, urban residence, higher ARCO stage, and smoking as the independent risk factors. However, the single- of its results, and their interpretation of certain factors appeared somewhat strained (6); for example, they highlighted the mental issues of patients with ARCO stage IV as a contributor for readmission, but the relevant data were not available.

Therefore, this study aims to investigate the incidence, primary causes, and risk factors for unplanned readmissions within one year after THA for ONFH, using data from two tertiary hospitals.

## Methods

### Inclusion and exclusion criteria

This study enrolled patients aged 18 years or older who had undergone their first primary THA for ONFH in two tertiary hospitals (Shijiazhuang People's Hospital and Third Hospital of Hebei Medical University) between January 2021 and December 2022 with a one-year follow-up. Patients who underwent simultaneous bilateral THAs or other surgeries, were tumor carriers, had missing data on medical records, operative records or imaging, or had incomplete follow-ups were excluded from the study. The decision to include the first primary THA was made to maintain consistency and reduce confounding in our analysis, facilitating the interpretation of the results. In cases of multiple readmissions within one year, only the first readmission was analyzed, with aims to simplify data interpretation, focus on initial complications, reduce related biases, and gain clearer insights for clinical practice.

### Demographic and clinical data

Clinical data during hospitalization were retrospectively collected for analysis. These included demographic information such as gender, age, BMI (kg/m^2^), place of residence, and comorbidities [e.g., chronic obstructive pulmonary disease (COPD), liver disease, heart disease, peripheral vascular disease, hypertension and diabetes], smoking and drinking status, Charlson Comorbidity Index (CCI), American Society of Anesthesiologists (ASA) classification, laboratory test results on admission (albumin, fasting blood glucose, red blood cell count, white blood cell count, neutrophil count, lymphocyte count, platelet count, hemoglobin, sodium ion concentration, potassium ion concentration, and D-dimer), surgical details including operative times, intraoperative blood loss, and anesthesia method, ARCO staging, perioperative blood transfusion, surgeon experience (measured by the number of THA cases since practice, and inexperience was defined as <20 cases) (10) and length of hospital stay.

Data collection was carried out by two trained research assistants, while the ARCO staging of ONFH was determined by the experienced leading surgeons. Patient information was sampled and verified by a trained research associate.

### Surgical procedure

All procedures were performed using the posterolateral approach. The patient was placed in the lateral decubitus position. A 10 cm curved incision was made on the posterior-lateral aspect of the affected hip. Layers of tissue were incised, including the skin, subcutaneous tissue, and fascia. The gluteus maximus muscle was cut along its fibers toward the greater trochanter until below the great trochanter. The affected limb was internally rotated to expose the external rotator muscle group, which was then cut along its attachment. The joint capsule was exposed and incised. The hip joint was dislocated posteriorly, and the femoral head was osteotomized based on the preoperative templating to restore the limb length. The joint capsule and synovial tissue were removed along the acetabular margin. The acetabulum was gradually enlarged and deepened using an acetabular reamer. Once the appropriate size was confirmed, the acetabular cup was implanted and secured with two screws, followed by the insertion of a liner. The limb was internally rotated, and the hip and knee were flexed to expose the femoral neck. The medullary cavity was gradually enlarged using a femoral rasp, and the femoral stem was inserted. The femoral head was installed, and the joint was reduced. Joint flexion and extension movements were checked for normal activity and stability. After confirming that the instruments and dressings were correct, the incision was washed and closed layer by layer.

Postoperatively, anticoagulation prophylaxis was routinely administered, typically involving low molecular weight heparin (LMWH), which was started on the night of the surgery and continued for 30 days postoperatively. Typically, patients stay in hospitals for 3 to 5 days after surgery, depending on recovery and complications. In the first 2 weeks, they are encouraged to start non-weight-bearing exercises as quickly as tolerated to improve joint motion; from 2 to 6 weeks, they may gradually begin partial weight-bearing exercises with support, focusing on balance to avoid falls; after 6 weeks, under physician guidance, they can progressively increase weight-bearing capacity while strengthening the hip and lower limb muscles, with timeline varying by individual condition.

### Outcome measure and follow-up

The outcome measure was defined as unplanned readmissions within 12 months after surgery due to any unexpected clinical medical or surgical events, whether related to the index THA procedure or not. Patients who were readmitted were determined by reviewing medical records and checking their hospitalization information. To prevent potential omission, especially for those readmitted to other institutions, we also contact them by telephone.

### Statistical analysis

Patients were classified into “readmission group” and “non-readmission group”, based on whether they experienced readmission. Continuous data were expressed with means (standard deviation, SD) and compared using Student-t test if normally distributed, or the Whiteny-U test if skewed. Categorical variables were expressed as numbers and percentages, and were compared using the Chi-square test. Variables tested with a *P* < 0.10 in the univariate analyses were further analyzed using a multivariate logistic regression model, employing the stepwise backward method for variable selection. Variables retained in the final model met a criterion of *P* value < 0.10. The magnitude of association between variables with the outcome was expressed as the odds ratio (OR) with 95% confidence interval (CI). To prevent overfitting of the multivariate model, the Hosmer-Lemeshow goodness-of-fit test was conducted, with results considered acceptable if *P* > 0.05 and adjusted Nagelkerke *R*^2^ < 0.750 (11).

The statistical significance level was set to 0.05. All statistical analyses were performed with SPSS 26.0 (IBM Corporation, NY, USA).

## Results

### General cohort characteristics of patients

A total of 594 eligible patients were included in this study, of whom 363 (61.1%) were men, with an average age of 59.2 ± 11.4 years at the time of surgery. Among them, 253 (42.6%) patients were aged 60 years or older. ONFH was present on the left side in 288 (48.5%) patients and on the right side in 306 (51.5%). According to the ARCO staging system, 209 (35.2%) cases were classified as stage III, and 385 (64.8%) as stage IV. Surgeries were performed at a median of 2 days after admission, with 542 (91.2%) within 3 days. A total of 26 surgeons were involved in the procedures. The average length of hospital stay was 12.7 ± 3.5 days.

### Characteristics of readmitted patients

Within one year after discharge, there were 47 cases (7.9%) of unplanned readmissions, among which 18 (38.3%) occurred within 30 days and 27 (57.4%) within 90 days. The readmitted patients had a median age of 66.0 (IQR, 57.0–72.5), and 61.7% were over 60 years old, predominantly being men (66.0%). The primary causes of readmission were mostly related to the index surgery, including hip dislocation (11, 23.4%), periprosthetic fracture (5, 10.6%), symptomatic deep venous thrombosis (DVT) of the lower extremity (6, 12.8%), delayed incisional union (4, 8.5%), deep infection (2, 4.3%), and persistent pain around the surgical site (4, 8.5%); there were also medical complications, including pneumonia (4, 8.5%), ischemic heart disease (5, 10.6%), urinary disease (4, 8.5%), ischemic stroke (2, 4.3%).

### Univariate and multivariate analyses for between-group differences

The readmission group had a significantly higher median age (median, 66.0; IQR, 57.0–72.5) compared to the non-readmission group (median, 58.5; IQR, 51.0–66.0) (*P* < 0.001), as well as a higher proportion of patients aged ≥60 years (*P* = 0.006). The average length of index hospital stay for readmitted patients was 16.6 ± 3.8 days compared to 12.4 ± 4.0 days for those without readmission (*P* < 0.001). In addition, the readmission group was significantly more likely to be obese (25.5% vs. 13.7%), classified as ASA Ⅲ-Ⅳ (21.3% vs. 11.3%), have heart disease (19.1% vs. 7.1%), have a CCI **≥4** (12.8% vs. 3.5%), present with anemia at admission (57.4% vs. 40.0%), and showed a tendency toward a higher prevalence of COPD (12.8% vs. 5.7%), albumin < 35 g/L (50.0% vs. 28.1%), and lower sodium ion concentration (23.4% vs. 13.2%) (Table 1).

**Table 1:** Univariate analyses of clinical and demographic characteristics for between-group comparisons.

Variables	Non-readmission group (*n* = 547)	Readmission group (*n* = 47)	*P* value
Age (years)	58.5 [51.0, 66.0]	66.0 [57.0, 72.5]	<0.001
≥60	224 (41.0%)	29 (61.7%)	0.006
<60	323 (59.0%)	18 (38.3%)	
Sex			0.478
Male	332 (60.7%)	31 (66.0%)	
Female	215 (39.3%)	16 (34.0%)	
Residence place			0.349
Rural	396 (72.4)	37 (78.7)	
Urban	151 (27.6)	10 (21.3)	
BMI (kg/m^2^)			0.029
<30.0	446 (86.3%)	35 (74.5%)	
≥30.0	71 (13.7%)	12 (25.5%)	
Sidedness			0.587
Left	267 (49.34)	21 (50.00)	
Right	280 (50.66)	26 (50.00)	
ARCO staging			0.149
Ⅲ	197 (36.0)	12 (25.5)	
Ⅳ	350 (64.0)	35 (74.5)	
ASA classification			0.045
Ⅰ-Ⅱ	485 (88.7)	37 (78.7)	
Ⅲ-Ⅳ	62 (11.3)	10 (21.3)	
Hypertension	139 (25.4%)	17 (36.2%)	0.108
Diabetes	57 (10.4%)	8 (17.0%)	0.164
Chronic obstructive pulmonary disease	31 (5.7)	6 (12.8)	0.053
Heart disease	34 (7.1%)	9 (19.1%)	0.004
Liver disease	21 (3.8%)	3 (6.4%)	0.395
Peripheral vascular disease	17 (3.1%)	2 (4.3%)	0.688
CCI ≥4	19 (3.5)	6 (12.8)	0.002
Current smoking	117 (21.4)	12 (25.5)	0.509
Current alcohol consumption	149 (11.3)	14 (6.2)	0.707
Surgeon inexperience (<20 cases since practice)	64 (11.7)	13 (27.7)	0.002
Anesthesia method			0.770
General	429 (78.4)	36 (76.6)	
Regional	118 (21.6)	11 (23.4)	
Operative times >120 min	122 (23.8)	13 (34.4)	0.400
Intraoperative blood loss >300 ml	132 (24.1)	13 (27.7)	0.589
Perioperative blood transfusion	52 (9.5)	4 (8.5)	0.823
Hospital stay (days)	12.4 ± 4.0	16.6 ± 3.8	<0.001
Anemia at admission	219 (40.0%)	27 (57.4%)	0.020
WBC count >10 × 1,012/L	43 (7.9%)	6 (12.8%)	0.241
Platelet count >300 × 109/L	40 (7.3%)	3 (6.4%)	0.813
Albumin <35 g/L	136 (28.1%)	17 (50.0%)	0.089
Fasting blood glucose >6.1 mmol/L	137 (25.0%)	14 (29.8%)	0.474
Sodium ion concentration < 135 mmol/L	72 (13.2%)	11 (23.4%)	0.052
Potassium ion concentration <3.5 mmol/L	60 (15.1%)	8 (25.0%)	0.211

Anemia was diagnosed according the hemoglobin concentration at admission with different reference ranges for females and males: male, <120 g/L; female, <110 g/L. BMI, body mass index; WBC, white blood cell; CCI, Charlson Comorbidity Index; ASA, American Society of Anesthesiologists; ARCO, Association Research Circulation Osseous.

The multivariate regression model showed age (every 10-year increment, OR, 1.39; 95% CI, 1.12–1.88), ARCO stage IV. vs. Ⅲ (OR, 3.72; 95% CI, 1.96–7.22), CCI ≥ 4 vs. < 4 (OR, 5.49; 95% CI, 2.16–13.77), hemoglobin < 100 g/L (OR, 2.72; 95% CI, 1.37–6.83) and surgeon inexperience (OR, 2.74; 95% CI, 1.29–6.73) were significantly associated with unplanned readmission (Table 2).

**Table 2:** Multivariate results for unplanned readmission after THA for ONFH.

Variable	*β* (coefficient)	Standard error	OR (95% CI)	*P* value
Age (each 10-year increment)	0.33	0.13	1.39 (1.12–1.88)	0.015
ARCO stage IV vs. Ⅲ	1.31	0.33	3.72 (1.96–7.22)	0.002
CCI ≥4 vs. <4	1.70	0.47	5.49 (2.16–13.77)	<0.001
Anemia on admission	1.00	0.41	2.72 (1.37–6.83)	0.026
Surgeon inexperience	1.01	0.42	2.74 (1.29–6.73)	0.027

ONFH, osteonecrosis of the femoral head; THA, total hip arthroplasty; OR, odds ratio; ARCO, Association Research Circulation Osseous; CCI, Charlson Comorbidity Index.

## Discussion

Understanding the clinical epidemiological characteristics of readmission after THA for ONFH is crucial for optimizing patient outcomes and minimize healthcare costs during perioperative management. In this study, we further explore this issue within the context of two tertiary hospitals. We found a one-year readmission rate of 7.9%, primarily related to the major surgical complications (ie, hip dislocation, periprosthetic fractures and symptomatic DVT of lower extremity). Advanced age, higher ARCO stage, severer comorbidity (CCI ≥4), admission anemia and surgeon inexperience were identified as significant independent risk factors.

The 30-day period post-discharge is critical for the medical and surgical stabilization of patients who have undergone major surgery. Our findings indicate that approximately 40% of readmissions occurred within this timeframe, involving both major surgical complications and a significant number of medical complications. This finding aligns with a recent study by Wang et al. (6), who observed 39.0% of readmissions occurring within 30 days after discharge. This suggests that surgeons should encourage patients to engage in specific exercises aimed at enhancing neuromuscular coordination, muscle strength, and balance, while also emphasizing the importance of diligent wound care to prevent complications related to the surgery. Additionally, patients should be advised to manage their underlying medical conditions effectively to reduce the risk of adverse events triggered by surgical trauma.

Advanced age (in 10-year increments) was associated with a 39% increased risk of readmission, highlighting the importance of need for intensified perioperative care for elderly patients (12). The underlying mechanism may involve a reduced functional reserve of organs, leading to decreased tolerance to surgical trauma and disturbances in internal homeostasis, thereby increasing the risk of medical complications (7). Additionally, older patients who undergo major musculoskeletal surgery are predisposed to having a significant declines in muscle strength and neuromuscular coordination, which heightens the risk of falls, fractures, and joint dislocations (13). This finding was consistent with a prior study of total hip or knee arthroplasty, which reported a 2-fold higher readmission rate in patients aged 61 to 70 years than those aged 51 to 60 years (3.8% vs. 1.9%) (12). Another study focusing on risk factors for readmission after THA, conducted by Paxton et al. (14), noted a 3% risk increase readmission at 30 days for each additional year of age.

Having multiple comorbidity (CCI ≥4) and anemia upon admission were also identified as strong risk factors for readmission. Furthermore, comorbidities significantly increased the risk of a variety of complications, including surgical delays, surgical site infection, and even mortality (15–17). Accordingly, surgeons should proactively evaluate and optimize the medical conditions of patients, especially elderly patients with greater medical complexity, provide tailored guidance on postoperative rehabilitation exercises and ensure close follow-up and monitoring to improve patient prognosis.

Multiple studies have confirmed that patients with advanced stages of ONFH tend to experience more extensive surgical trauma, poorer functional recovery, and increased complication rates. This can be attributed to several factors, including older age, more severer comorbidities, and greater complexity of bone and soft tissue conditions (18–20). On one hand, patients with ARCO Ⅳ ONFH often exhibit significant damage to joint surfaces, alterations in acetabular bone, and secondary soft tissue stiffness, necessitating longer operative times and more extensive incisions for precise component positioning (21). On the other hand, patients with ARCO stage Ⅳ ONFH often reduce their activity levels or resort to long-term bed rest due to joint pain, which increases the risk of cardiovascular events, pneumonia, and deep vein thrombosis in the lower extremities. In a prospective study conducted by Wang et al. involving 876 ONFH patients, ARCO stage Ⅳ was associated with significantly poorer outcomes compared to stage III, including longer operative times, greater blood loss, higher one-year readmission rate and overall complication rate, and poorer functional outcomes as measured by the Harris hip score and higher dislocation rate (19). Therefore, undergoing THA at stage III may be a preferable option.

Given the complexity of the THA procedure, achieving proficiency requires a substantial learning curve in precise component positioning, alignment, and soft tissue management, all of which greatly influence postoperative outcomes. In this study, we found that surgeon inexperience with THA was associated with a moderate-intensity risk of readmission (OR, 2.74), and the patients operated on by the inexperienced surgeon had a readmission rate of 16.9%, significantly higher than the 6.6% for those treated by the experienced surgeons (6.6%). A recent systematic review and meta-analysis comparing various THA approaches concluded that “choice of THA approach should ultimately be guided by surgeon experience” (22). Given the demands of the posterolateral approach primarily utilized in our study, which necessitates advanced surgical techniques and skills, we recommend referring complex cases—particularly those complicated by medical instability or challenging local surgical conditions—to experienced surgeons whenever possible.

There are several limitations to this study. Firstly, its retrospective nature and reliance on medical records for data collection may have introduced recall and measurement biases. Second, information on patient compliance with the postoperative rehabilitation protocol was not available, because it may be difficult for patients to accurately report their adherence. Third, residual confounding remains due to the inability to capture many clinical data, e.g., the etiology and duration of ONFH, medications usage, osteoporosis, preoperative functional scores, postoperative exercises (frequency and intensity). Further studies should continue to address these limitations. Fourth, despite being conducted at two tertiary and university-affiliated centers, it remains unclear whether these findings can be generalized to other settings or populations.

In conclusion, this study revealed a 7.9% rate of unplanned readmission following THA in patients with ONFH. It also identified the primary causes of readmission and the independent risk factors associated with it. These findings provide clinical insights into this important clinical event and can enhance preoperative counselling for patients and improvement of perioperative care.

## Data Availability

The data analyzed in this study is subject to the following licenses/restrictions: In accordance with institutional policy for patients' medical records, data used in this study are not open publicly, but will be obtained upon motivated request to the corresponding author for purpose of scientific research. Requests to access these datasets should be directed to Xuewei Hao, haoxuewei1981@163.com.
